# African swine fever: a global view of the current challenge

**DOI:** 10.1186/s40813-015-0013-y

**Published:** 2015-12-23

**Authors:** Ma Carmen Gallardo, Ana de la Torre Reoyo, Jovita Fernández-Pinero, Irene Iglesias, Ma Jesús Muñoz, Ma Luisa Arias

**Affiliations:** 1 European Union Reference Laboratory (EURL) for African swine fever, INIA-CISA, 28130 Valdeolmos, Madrid Spain; 2 FAO Reference Centre for African swine fever, INIA-CISA, Valdeolmos, 28130 Madrid Spain; 3 Epidemiology Department, National Institute for Agricultural and Food Research and Technology, Animal Health Research Centre, INIA-CISA, 28130 Valdeolmos, Madrid Spain

**Keywords:** African swine fever, Aetiology, Epidemiology, Clinical symptoms, Transmission, Diagnosis, Prevention, Control strategies

## Abstract

African Swine Fever (ASF) is an important contagious haemorrhagic viral disease affecting swine whose notification is mandatory due to its high mortality rates and the great sanitary and socioeconomic impact it has on international trade in animal and swine products.

This disease only affects porcine species, both wild and domestic, and produces a variety of clinical signs such as fever and functional disorders of the digestive and respiratory systems. Lesions are mainly characterized by congestive-haemorrhagic alterations. ASF epidemiology varies significantly between countries, regions and continents, since it depends on the characteristics of the virus in circulation, the presence of wild hosts and reservoirs, environmental conditions and human social behaviour. Furthermore, a specific host will not necessarily always play the same active role in the spread and maintenance of ASF in a particular area.

Currently, ASF is endemic in most sub-Saharan African countries where wild hosts and tick vectors (*Ornithodoros*) play an important role acting as biological reservoirs for the virus. In Europe, the disease has been endemic since 1978 on the island of Sardinia (Italy) and since 2007, when it was first reported in Georgia, in a number of Eastern European countries. It is also endemic in certain regions of the Russia Federation, where domestic pig and wild boar populations are widely affected. By contrast, in the affected eastern European Union (EU) countries where ASF is currently as epidemic, the on-going spread of the disease affects mainly wild boar populations located in restricted areas and, to a much less extent, domestic pigs. Unlike most livestock diseases, no vaccine or specific treatment is currently available for ASF. Therefore, disease control is mainly based on early detection and the application of strict sanitary and biosecurity measures. Epidemiology of ASF is very complex by the existence of different virus circulating, reservoirs and a number of scenarios, and the on-going spread of the disease through Africa and Europe. Survivor pigs can remain persistently infected for months which may contribute to virus transmission and thus the spread and maintenance of the disease, thereby complicating attempts to control it.

## Introduction

**Fig. 1:**
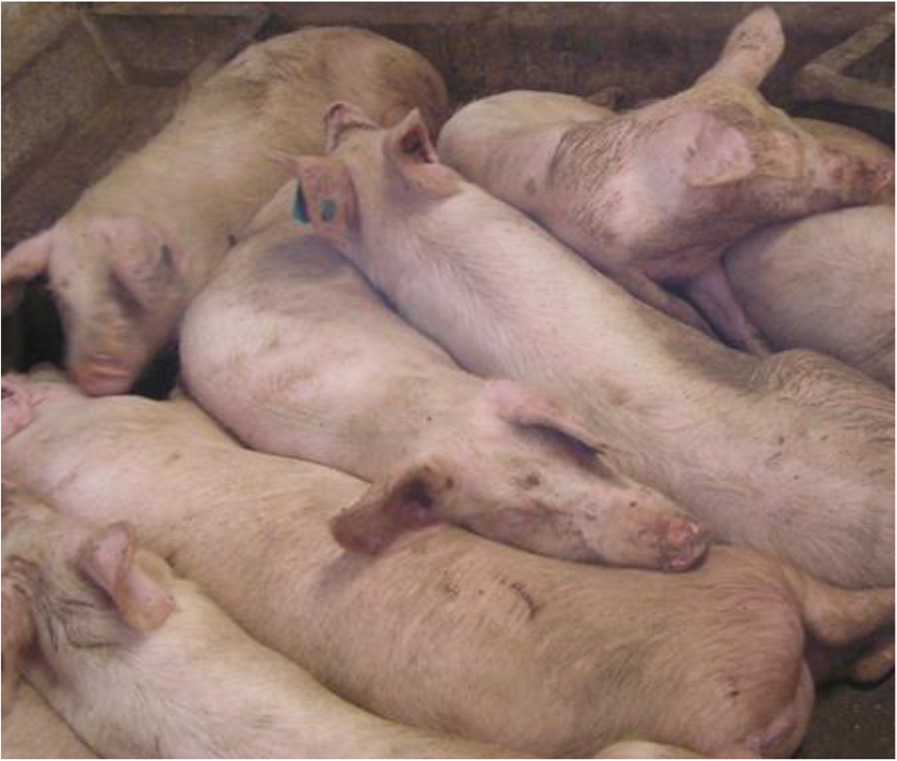
Clinical signs of acute form of ASF (source: EURL, INIA-CISA, Valdeolmos, Madrid, Spain). Pigs affected by acute form of ASF showing prostration and reddening of the skin at the tips of ears

African swine fever (ASF) is one the most important of all swine diseases due to its significant sanitary and socioeconomic consequences. Infected animals show a wide variety of clinical forms and lesions that vary in terms of the virulence of the virus and the immunological characteristics of the host. Acute forms are predominant at the beginning of outbreaks in disease-free areas resulting in high mortality rates of up to 95–100 %. Figure 1 Once established, the disease progresses towards its acute and subacute clinical forms that are sustained over time, although other clinical forms (chronic and subclinical or unapparent) will eventually evolve in regions where the disease is endemic.

**Fig. 2:**
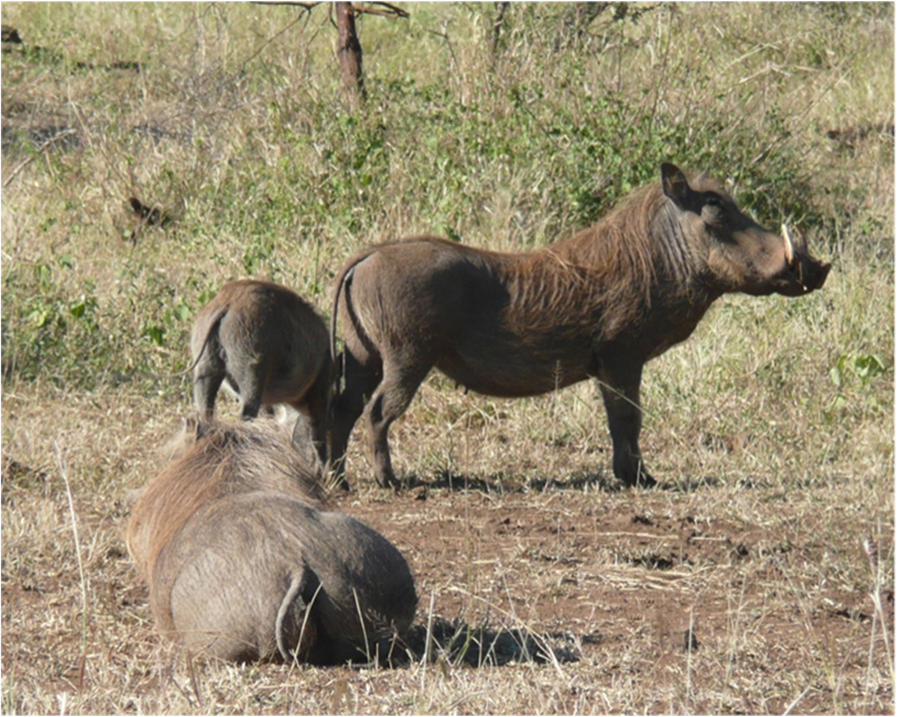
Warthog (*Phacochoerus africanus*). *Phacochoerus* genera act as the reservoirs of the ASFV in Africa without clinical symptoms. Transmission and maintenance of ASFV can occur in a sylvatic cycle involving warthogs and bushpigs as well as ticks of the genus *Ornithodoros*

European wild boar (*Sus scrofa*) and feral pigs are very susceptible to the disease and exhibit similar clinical signs and lethality to domestic pigs. By contrast, infected wild African Suidae develop subclinical and asymptomatic long term persistent infections, acting as virus reservoirs [1]. Figure 2 Soft ticks of the genus *Ornitodoros* also act as biological vectors and reservoirs for the virus [2, 3].

**Fig. 3:**
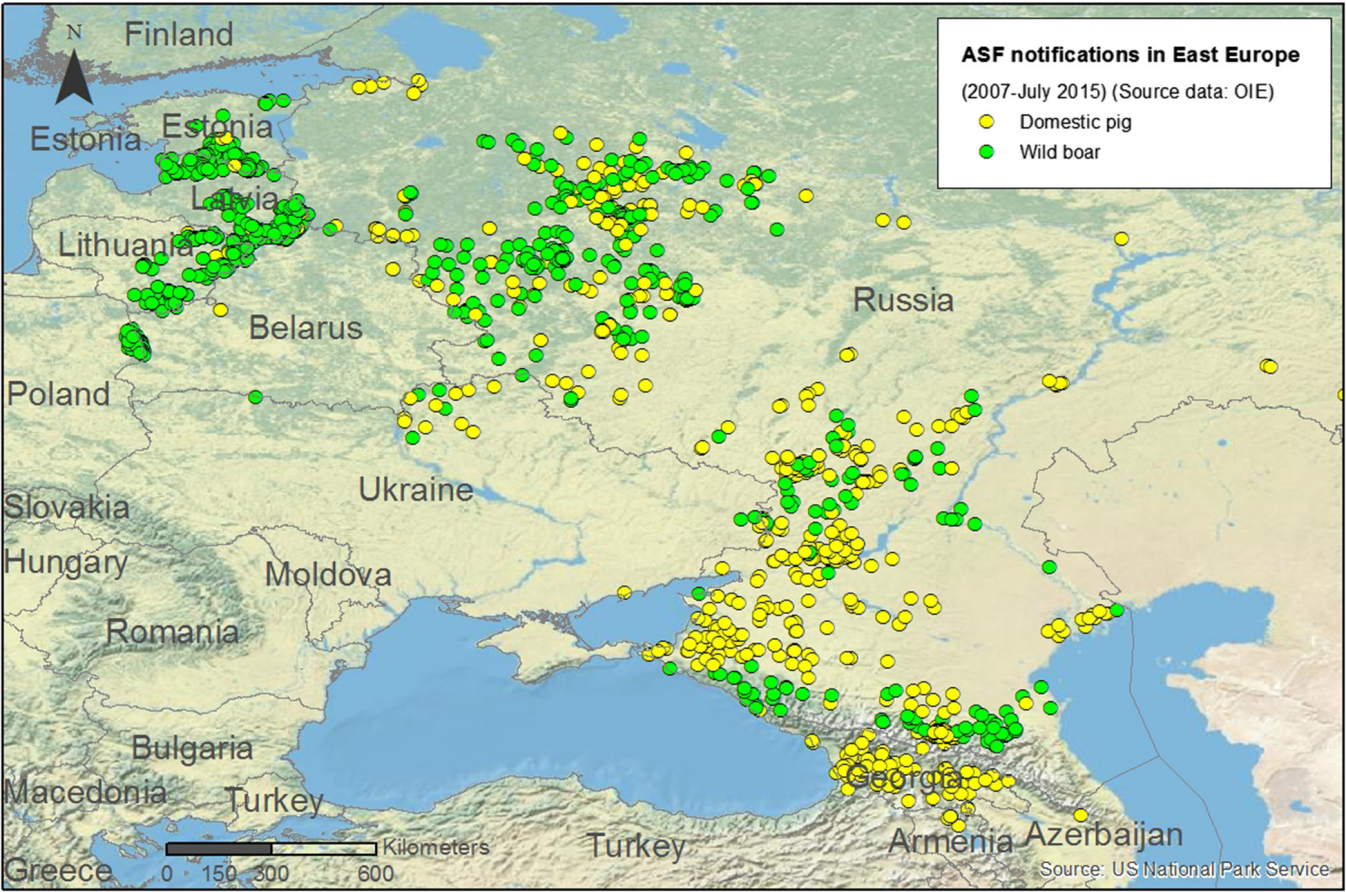
ASF notifications in Eastern Europe (source: A. Rodríguez (INIA-CISA, Valdeolmos, Madrid, Spain). Geographic map showing notifications of ASF in Eastern Europe since 2007 to July 2015. In green dot notifications in European wild boar. In yellow dot notifications in domestic pigs. Source: OIE

ASF is endemic in most sub-Saharan countries. Since it was introduced in 2007 into Eastern Europe it has affected the Caucasus region and the Russia Federation, where it now exists as a large-scale epidemic in domestic pig and wild boar populations in two endemic zones in central and southern Russia [4, 5]. This situation, together with the recent incursions of the disease into the European Union (Fig. 3) and its complexity, underlines the need to clarify some of the important uncertainties regarding the epidemiology of ASF – for example, how the virus is transmitted and how virus-host interactions are established-in order to implement effective control-eradication strategies. The role of wild and domestic hosts in the different scenarios, the importance of environmental, social and cultural factors, and the part played by survivor pigs are just some of the important gaps in our knowledge that need urgently to be filled. The current situation of ASF in Africa and Europe is today a major threat to the pig industry worldwide.

### History

**Fig. 4:**
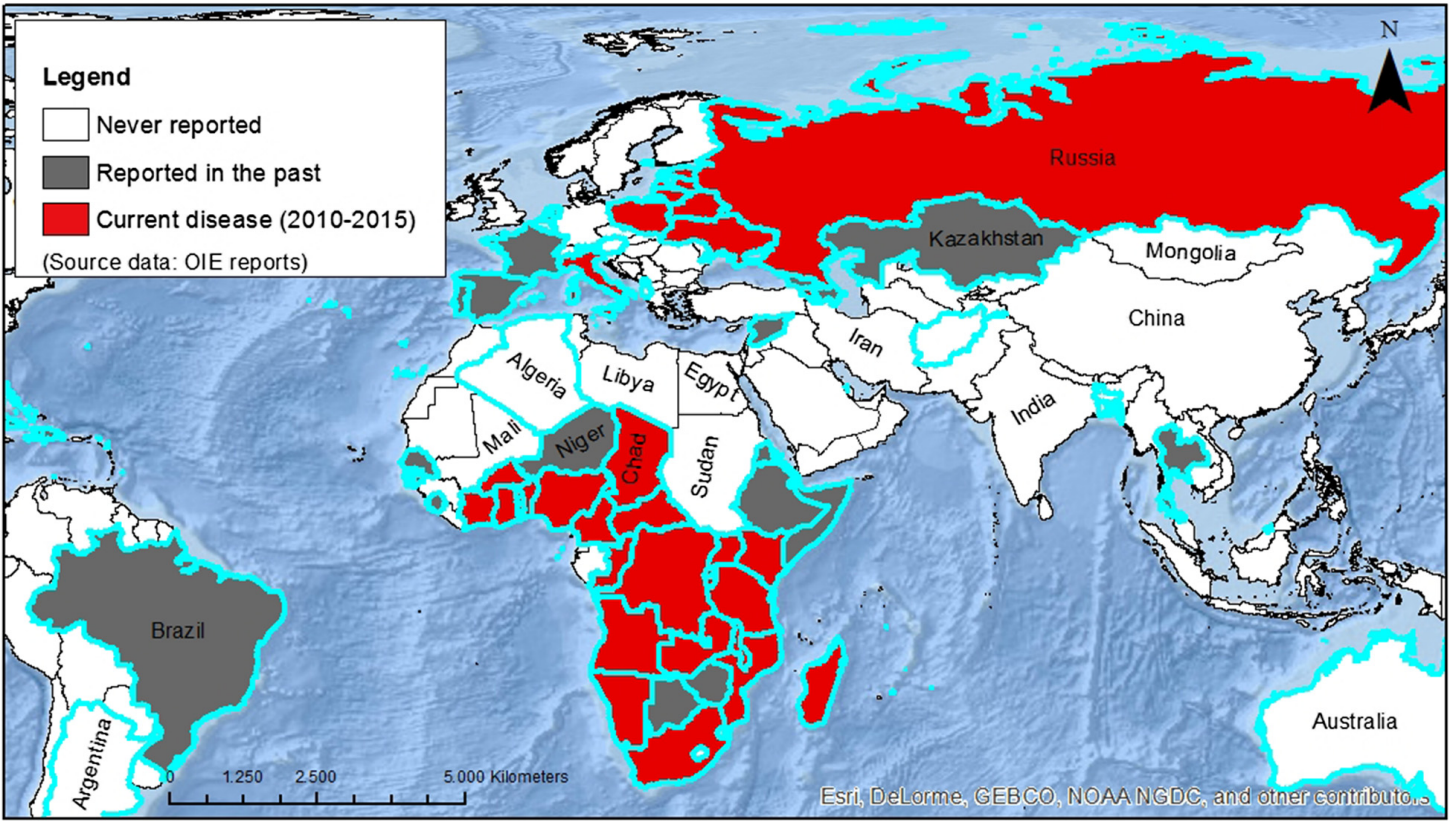
Geographic distribution of ASF worldwide. In red, countries in which ASF is currently present from 2010 to date. In grey, countries in which ASF was reported in the past. In white, countries in which ASF has not been never reported

ASF was first detected in Kenya in 1909 following the introduction into the country of European domestic swine. It was reported as an acute haemorrhagic disease with mortality rates of 100 % in domestic pigs [6, 7]. It was then recognised that the disease had been present in eastern and southern Kenya in wild hosts for a very long time. Subsequently, it was detected in Central and West Africa but was confined to sub-Saharan African countries until it first reported outside Africa in 1957 in Lisbon (Portugal), from where it had spread from West Africa. After two years silence, in 1960 the disease appeared again in Lisbon and soon spread to the Iberian Peninsula and other countries in Europe such as France (1964), Italy (1967, 1969, 1983), Malta (1978), Belgium (1985) and the Netherlands (1986). Various countries in the Americas were also affected by ASF during this period: Cuba (1971, 1980), Brazil (1978), the Dominican Republic (1978) and Haiti (1979). In all these countries the disease has been successfully eradicated, the exception being the island of Sardinia (Italy) (Fig. 4).

### The virus

**Fig. 5:**
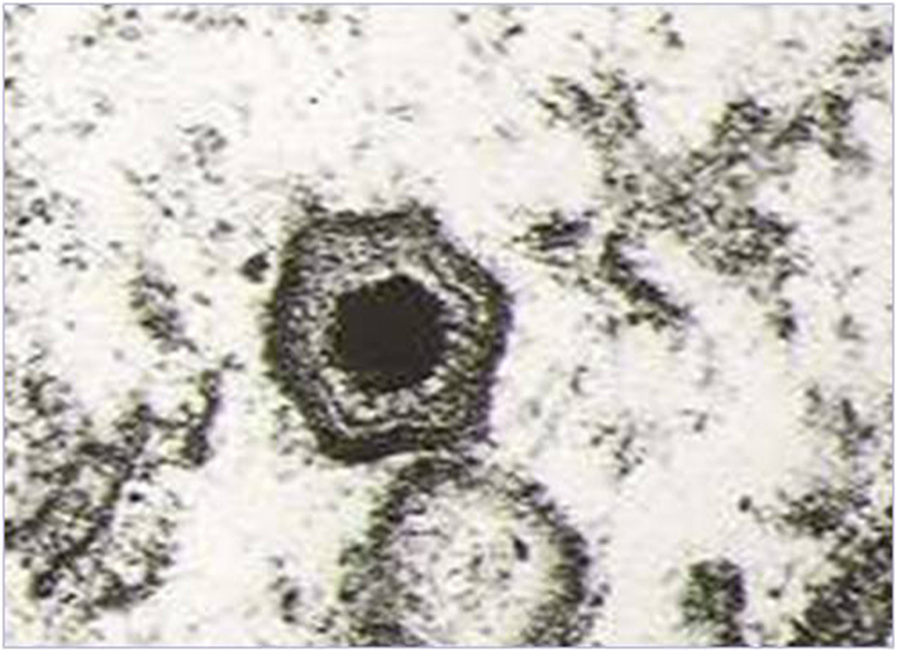
Electron micrograph of ASFV (source, INIA-CISA, Valdeolmos, Spain). By electronic microscopy, viral particles show an average diameter of 200 nm. The virion is formed by several concentric structures with an external hexagonal envelope. The main target cells for ASFV replication are monocytes and macrophage cells

The causative agent of the disease, the ASF virus (ASFV), is the only member of the Asfaviridae family, genus *Asfivirus* [8] (Fig. 5). It is a complex enveloped virus with icosahedral morphology consisting of four concentric layers and a large double-stranded DNA molecule that ranges in length between isolates from about 170 to 193 kbp [9]. It contains a conserved central region of about 125 kb and two variable ends. The differences in genome length are largely due to the gain or loss of members of the multigene families (MGF) located in the left and right variable regions [9]. A full genome sequencing of up to 16 virus isolates has recently been completed [10–14]. The ASF viral DNA contains between 151 and 167 open reading frames (ORFs) encoding 54 structural proteins and around 100 polypeptides in the targeted infected cells, monocytes and macrophages [15, 16]. The major components of the viral capsid, the protein p72, the two structural proteins p30 (p32) and p54 and the polyprotein pp62, have been identified as the most antigenic of the proteins that are responsible for the induction of antibodies after a natural infection [17, 18]. However, despite the usefulness of these proteins as sero-diagnostic targets, they are not sufficient for developing antibody-mediated protection against virus strains [19].

### Inactivation of ASFV

Although ASFV is very resistant to inactivation in the environment, many lipid solvents and commercial disinfectants based on phenolic and iodide compounds are effective and can inactivate the virus at pH < 4 and pH >11 [20].

The virus may persist for several weeks or months in frozen, fresh or uncooked meat, as well as in salted dried meat products [20]. By contrast, ASFV is inactivated in cooked or canned hams when these products are heated to 70 °C and in cured or processed products such as Spanish cured pork products (e.g. *serrano* and Iberian hams and shoulders) at day 122–140 of curing [21].

### Virus genotyping

Recent studies have reported a classification of 32 ASFV isolates in eight different serogroups based on a hemadsorption inhibition assay (HAI) with ASFV reference immune antisera [22].

**Fig. 6:**
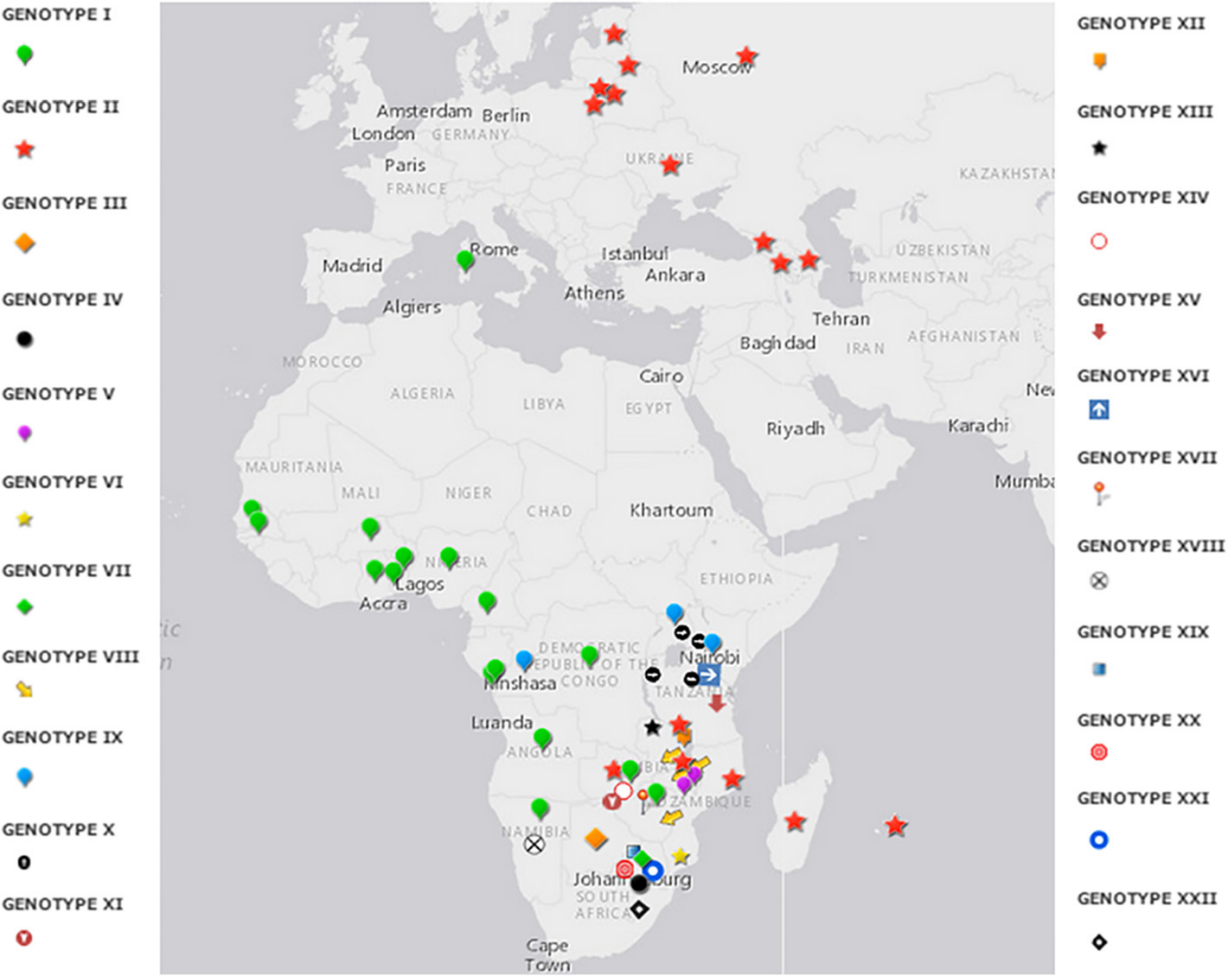
Distribution of ASF virus genotypes (source: EURL, INIA-CISA, Valdeolmos, Madrid, Spain). Symbols represent the 22 AFSV genotypes determined by partial *B646L* (p72) sequencing known to be in circulation within that country. Genotypes are indicated in roman numerals

However, despite conventional ASFV genotyping cannot discriminate between viruses of different virulence, it has been widely demonstrated throughout more than 10 years that the molecular characterization of small conserved regions of the DNA genome is the most useful tool for tracing the origin of ASFV during outbreaks [23–39]. The current approach is based on the analysis of the C-terminal end of gene *B646L* encoding the major protein p72 [39], following by the sequencing of the Central Variable Region (CVR) within the *B602L* gene, or other several regions (e.g. *E183L* encoding p54 protein, *CP204L* encoding p30 protein), for distinguishing between geographically and temporally constrained p72 genotype viruses [28–30, 33, 34, 36, 38]. This approach has allowed identifying twenty-two different p72 genotypes among virus isolates from East and South African countries to date, whereas genotype I is predominant in West Africa [35, 37]. Outside Africa, genotype I was the only one found in Europe, America, and the Caribbean, until the introduction of genotype II in 2007 into Georgia in 2007 from East Africa (Fig. 6) [33]. Current available molecular data derived by using standardized genotyping procedures have indicated the presence of only p72 genotype II circulating in eastern European indicating a single introduction in 2007 [23, 27]. A variable region between genes characterized by the presence of tandem repeat sequences (TRS), recently identified as useful for subtyping gene identification, has revealed the presence of two variants in genotype II amongst the viruses circulating in Eastern European countries since 2012 [23, 40].

### Routes for virus entry, pathogenesis and transmission

The entrance of ASFV into pigs normally occurs either orally or nasally, although other routes such as cutaneous, subcutaneous, via tick-bites or scarification have also been reported. Traditionally, virus entrance into free regions usually occurs as a result of uncooked pork waste–especially from ships and aircraft–being fed to pigs. Once the disease is established in an area, it mainly spreads by direct contact between sick and healthy animals (domestic pigs and wild Suidae), recovered carrier pigs and soft ticks or, for example, through indirect transmission by lorries, at drinking and eating troughs, via surgical and personal equipment, rodents, or other farm animals [1].

ASF has an incubation period of 4–19 days. Clinical course lasts for 4–5 days in acute infections or longer in cases of the subacute forms of the disease. Usually, peracute and acute forms appear at the beginning of the epidemic, which is characterized by high lethality and the rapid spread of outbreaks [41, 42]. Once the disease is established as endemic in an area, a broad range of clinical symptoms and clinical onsets are to be expected, with an increasing number of subacute, chronic and subclinical infections but with mortality rates that decline over time. Infected animals can survive for several weeks and some even recover from the infection and remain sub-clinically infected for a period of time [41, 43–49]. In endemic zones, the disease progresses towards subacute and subclinical forms, sometimes due to the appearance of virus isolates of moderate and low virulence that are more difficult to recognize in the field. In these cases, the infection may persist for several months with no particular obvious symptoms in infected animals other than transient fever, stunting or emaciation, symptoms that may even mimic certain other diseases [42, 45, 50–55].

### Clinical symptoms and lesions

Unlike Classical Swine Fever (CSF), which mainly affects young pigs, all age groups are equally susceptible to ASF. ASFV strains are classified as of high, moderate or low virulence [55–58]. Highly virulent strains are usually responsible for the peracute and acute forms that provoke high mortality rates that may reach 100 % within 4–9 days post-infection. In peracute ASF, affected animals may die suddenly 1–4 days after the onset of clinical signs with no evident lesions in organs.

**Fig. 7:**
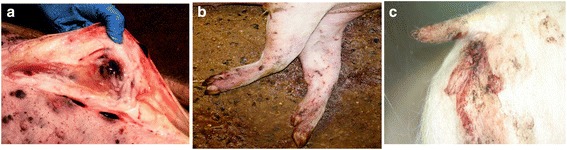
Clinical signs and lesions of acute form of ASF in a domestic pig experimentally infected with an ASFV genotype II isolate circulating in Eastern Europe (source: EURL, INIA-CISA, Valdeolmos, Madrid, Spain). **a** Necrotic areas on the skin surface, **b** subcutaneous haematomas in legs and **c** melena

**Fig. 8:**
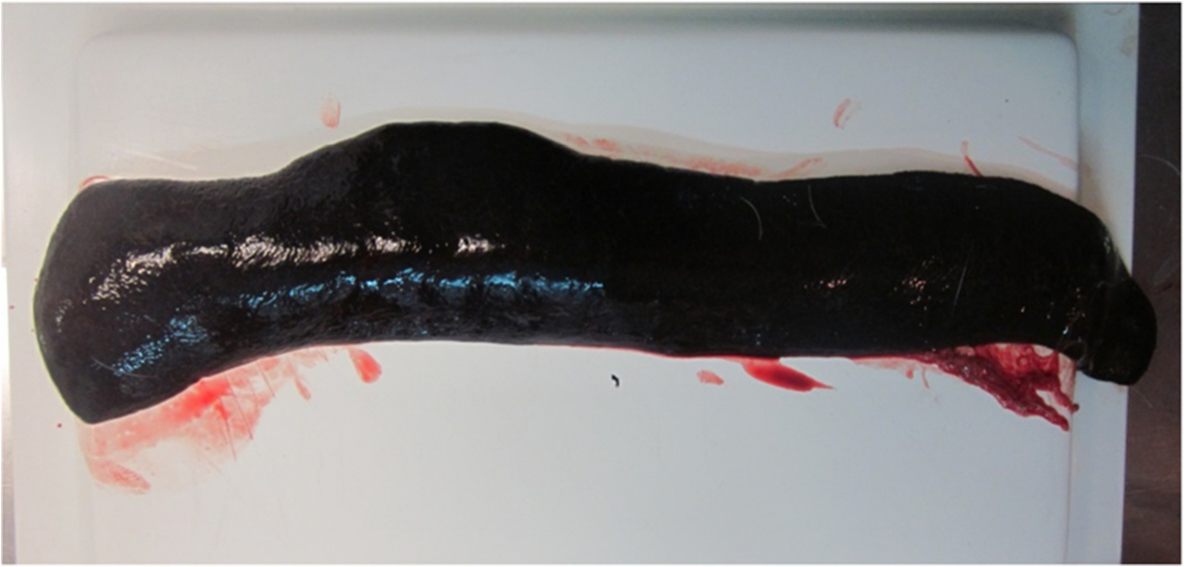
Gross lesions of acute form of ASF in a domestic pig experimentally infected with an ASFV genotype II isolate circulating in Eastern Europe (source: EURL, INIA-CISA, Valdeolmos, Madrid, Spain). Spleen displaying hyperemic splenomegaly (enlarged with rounded edges, friable and dark red to black)

**Fig. 9:**
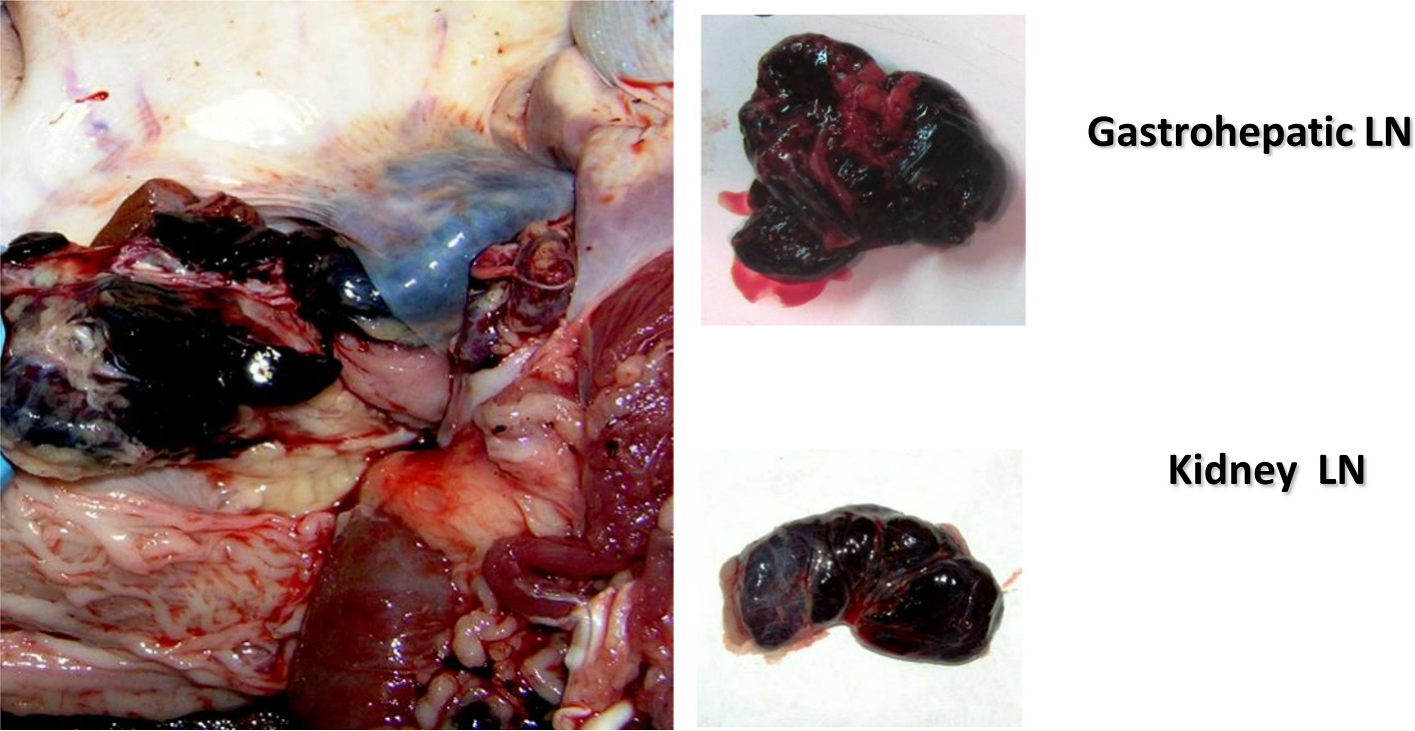
Gross lesions of acute form of ASF in a domestic pig experimentally infected with an ASFV genotype II isolate circulating in Eastern Europe (source: EURL, INIA-CISA, Valdeolmos, Madrid, Spain). Lymph nodes (LN) enlarged edematous and completely hemorrhagic similar to a blood clot, mainly gastro hepatic and kidney LNs

The **acute form** of the disease is usually characterized by a febrile syndrome with erythema and cyanosis of the skin (Fig. 7). Functional failures of internal organs, above all of the digestive system, vomiting and haemorrhagic diarrhoea may occur. Anorexia, cyanosis and incoordination may occur 1–2 days before death. Abortion in pregnant sows has frequently been described. Internal lesions are mainly characterized by hyperaemic splenomegaly and haemorrhages in organs, particularly in the visceral lymph nodes, with an excess of natural fluids in body cavities and spaces [55, 57, 59] (Figs. 8 and 9).

In **subacute forms** of the disease, a persistent or fluctuating fever lasts for up to 20 days; during this time, some pigs remain healthy, while others display the symptoms described above for the acute form, albeit less severely than normal. The mortality rates for subacute forms are in the range 30–70 % usually after 20 dpi. The subacute form is characterized by milder lesions than those described for the acute form [55].

In the **chronic form** of ASF, clinical signs and lesions are not specific but may persist for several months, giving rise to a range of conditions with symptoms such as skin ulcers and arthritis, delayed growth, emaciation, pneumonia and abortion. Overall, the clinical signs associated with the chronic form do not resemble the typical clinical picture of ASFV infections [7, 41, 43–45, 50, 55, 56, 60–62].

### The role of survivor pigs

Survivor pigs, sub-clinically infected and chronically infected, can remain persistently infected for months which may contribute to virus transmission and play an important role in disease persistence in endemic areas, as well as in sporadic outbreaks and ASFV introductions into disease-free zones [1, 48, 49, 63–65]. Studies of virus persistence and the transmission of ASFV to susceptible animals are scarce. *In vivo* experiments using European domestic pigs have revealed an infectious period of moderately virulent virus isolates ranging from 20 to 40 days [66]. Other *in vivo* study of ASFV transmission with a virus isolate of low virulence have shown that recovered pigs are still able to transmit the virus to naive populations three months after being infected [67]. Field studies performed in affected regions of Brazil and the Iberian Peninsula (1979–1981) have revealed that 3.5 % and 0.6 % of new outbreaks are thought to be caused by seropositive domestic pigs that have survived the initial infection [43, 47, 65]. Recent observations in endemic regions of East Africa such as Tanzania have estimated the presence of asymptomatic seropositive pigs at 3.72 %, even one year after the occurrence of an ASF outbreak [68]. Similar findings have been reported from Kenya and Uganda [69, 70].

The persistence of viruses in tissues of infected animals for up to six months has been repeatedly proven which indicates the length of the risk period during which the disease can be contracted from infected carcasses [51, 52, 71, 72].

### Some considerations regarding ASF in affected regions

A) A brief view of the disease in Africa
  The pig population in Africa is concentrated in sub-Saharan Africa, mostly on small-scale family farms. AU-IBAR-FAO data obtained in 2013 show it consisted of around 32 million heads distributed regionally as follows: 42, 32, 14 and 12 % in western, southern, eastern, and central Africa, respectively [73]. ASF was present in most of these areas in the past. However, from 1995 onwards, a significant increase in the number of ASF outbreaks occurred in sub-Saharan regions and new countries were affected. The disease spread extensively particularly in western regions and on some islands previously free of the disease, such as Madagascar and Mauritius. This upsurge of the disease in Africa, coupled with a lack of awareness, were crucial factors in the spread of the virus beyond Africa and into the Caucasus region (Georgia, Europe) in April 2007. As well, the disease recently re-emerged in 2014 and 2015 in the Ivory Coast and on Cape Verde, respectively, after over 15 years of silence (OIE notifications).
  To date, ASF is endemic in more than 23 countries in sub-Saharan Africa. The transmission of the ASF virus occurs via a number of complex epidemiological scenarios that depend on the presence of the presence of reservoirs, wild Suidae and soft ticks (*Ornithodoros moubata*), and domestic pig hosts, certain types of animal production and husbandry, and social behaviour. ln eastern and southern Africa, where all the 22 ASFV genotypes are known to circulate, the disease is maintained by the concurrent existence of transmission cycles involving asymptomatic wild Suidae (*Phacochoerus* and *Potamochoerus* spp.), soft ticks (*O. porcinus*) and domestic pigs. By contrast, the pig to pig transmission cycle is predominant in western Africa, where no ticks have been reported to date [1, 37, 49].
  The clinical picture of ASF reported in domestic pigs in African regions shows acute and sub-acute forms of the disease, associated with both virulent and moderately virulent virus isolates. In these animals, the viraemia starts few days after infection and antibody response can be usually detected from the second week post-infection onwards [62, 71]. In addition, the presence of subacute-to-unapparent clinical signs in local ‘indigenous’ domestic breeds in regions of East African has also been described in recent years. An *in vivo* experiment in both, local indigenous and European breeds, showed local indigenous pigs displayed a clear significant delay in the onset of ASF infection compared to the European breeds of pig, as well as an unclear, unspecific and non-pathognomonic clinical picture of the disease. High viraemia was detected in both groups, and a significant delay in the detectable antibody response was also observed, results that in some cases were absent in the local breed when compared to European breed [74].
B) A brief view of the disease in Eastern Europe
  Once introduced into the Caucasus in 2007, ASF spread rapidly into the neighbouring countries of Armenia and Azerbaijan, and then into southwest Russia, from where, due to the lack of any effective control measures, it continued its spread into the Ukraine (2012) and Belarus (2013). At present, a clear endemic pattern in both domestic pigs and wild boars has been identified in two regions of southwest and central Russia [5].
  The first notification of ASF in an EU country occurred in January 2014 in wild boar from Lithuania. A month later, new cases of ASF were detected in Poland and later in Latvia and Estonia. To date (09/2015), nearly a thousand of ASF notifications in wild boar and in lesser extent in domestic pigs (around 75, mostly in backyard farms) have been reported, in many cases in the eastern regions of the above-mentioned countries that border on Belarus and Russia. It is highly likely that the disease was introduced into the EU in 2014 by wild boar entering Lithuania, Poland and Latvia, from Belarus, or Estonia from Latvia [75].
  The ASFV isolates circulating in Eastern Europe are virulent viruses that induce **acute forms of ASF with high lethality in both domestic and wild animals** [59, 76–78]. Deaths usually occur in the second week after infection. However, since the introduction of the virus into the Russian Federation in 2007 ASF has become a large-scale epidemic involving both domestic pig and wild boar populations, with two recognized endemic zones in central and southern parts of the Russian Federation [5]. Field and experimental findings in Russia reveal the existence of sero positive wild boar animals previously diagnosed at the limit of the detection or negative when tested with the ASF virological assays [79–81]. These data suggest that, despite the virulent nature of current ASFV circulating strains affecting East Europe, some animals can survive for over a month and are able to recover from the infection, even remaining sub-clinically infected, and could become virus carriers enabling the virus to persist and spread amongst the porcine population [59, 67]. It has been demonstrated that in areas where ASF becomes endemic, increased numbers of subacute and subclinical infections also occur, and that mortality rates decline over time [43, 44]. This could be related to the acquired immunity from previous exposure to lower doses of virus, adaptation of the virus to the host and/or the evolution to viruses with reduced virulence which can emerge after many years of circulation into the pig populations [1, 7].
  It is important to note that, in contrast to Russia, where domestic pigs play a major role in the transmission of the disease [82, 83], to date in the EU wild boar are the hosts that are causing greatest concern. The ASF cases occurring in the wild boar population demonstrate the epidemiological complexity of the scenario and the inherent difficulties in containing the propagation of the virus in the regions bordering on Russia and Belarus. In these countries, the disease is not being effectively controlled and continued spill-over into bordering regions is likely to occur as a result.

### Understanding ASFV infection dynamics on industrial pig farms

Some outbreaks on pig farms have been particularly dramatic and thousands of animals have had to be slaughtered. Therefore, it is worth analysing how ASFV infections occur and what lessons should be learned from such outbreaks. A good example occurred during summer 2014 in the Ignalina region (Utena province) in northeast Lithuania, 22 km from the frontier with Belarus [84]. This outbreak occurred in an intensive industrial pig farm, with a closed cycle and very strict biosecurity measures, and had catastrophic consequences as over 20,000 animals had to be sacrificed. As a result of the observations it was demonstrated after its first entry in a pig farm, the virus moves and spreads initially without evident clinical signs of ASF. The first sign was the sudden death of a few animals in the same shed, which was associated to other causes, such as poisoning. Following this initial infection, the virus multiplied resulting in a second wave of infection within 12–14 days that leads to many more deaths. This second–or even third–wave affects more animals in the same area, and leads to devastating waves of deaths a few days later in which almost all the animals in the same stall die.

This information offers crucial insights: disease field recognition should be one of the major pillars of early disease detection. Therefore, tighter clinical controls must be established. In high-risk areas, sudden deaths of a few animals should not simply be attributed to common causes and should, instead, be treated with the upmost seriousness. It is important to point out that different evolution patterns of the ASFV infection can be expected. The movement and the spread of the waves of ASFV infection will depend on biosecurity conditions and the type of production system, husbandry, management and organization involved. A key element in prevention – above all in areas at risk either due to their proximity to infection sources or to trade routes – is to heed even minimal clinical symptoms, i.e. fevers, when they appear, even if they only affect just a few animals. Periodical clinical checking must be carried out, along with the implementation of strict biosecurity measures based on available protocols designed to control the movements of animals, staff and vehicles. The earlier the disease is detected, the fewer the losses and the easier it will be to halt the propagation of the virus to other farms.

### ASF Diagnosis

**Fig. 10:**
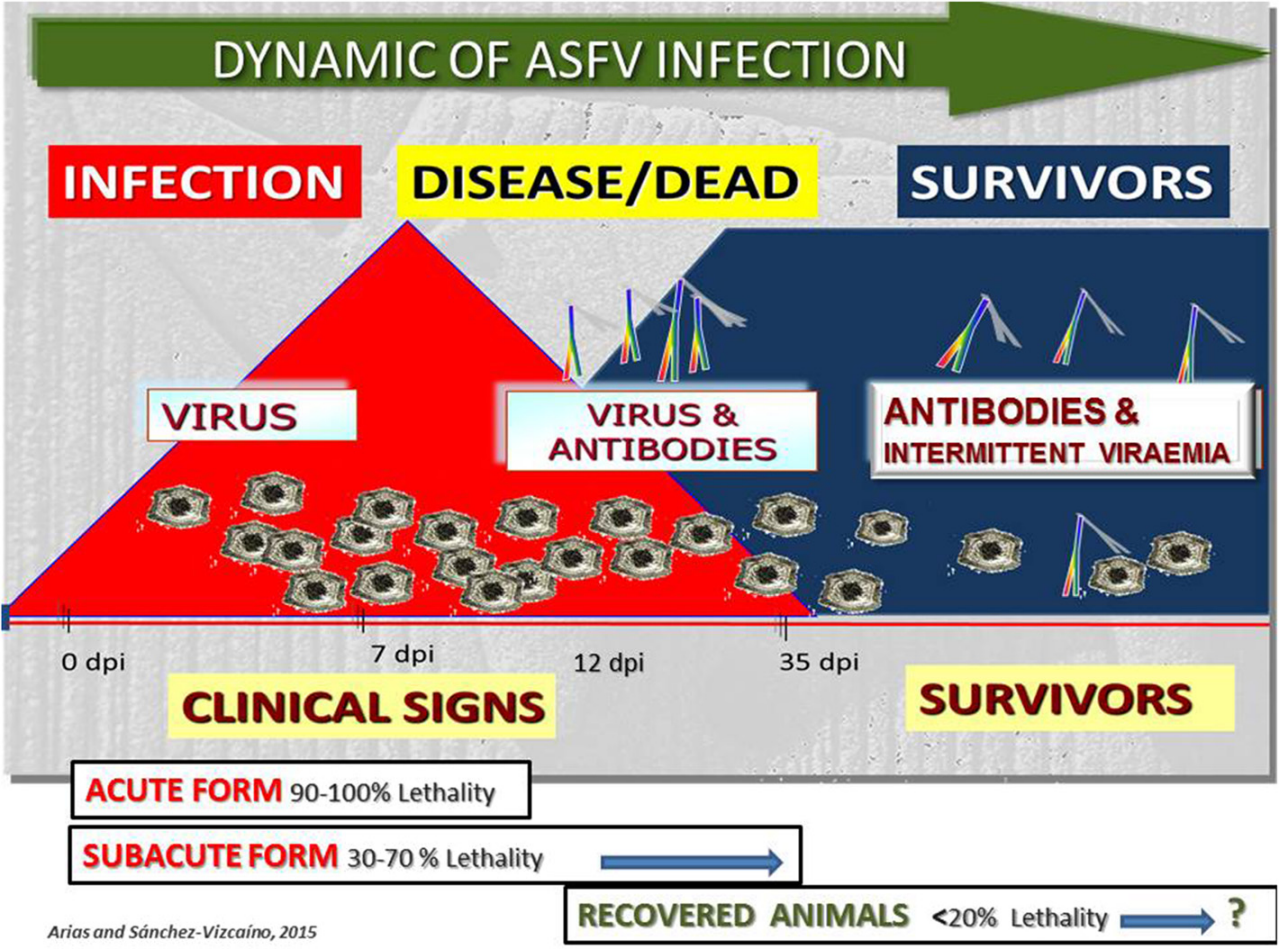
Dynamic of ASF infection (source: OIE WRL, UCM, Madrid, Spain and EURL, INIA-CISA, Valdeolmos, Madrid, Spain). The picture summarizes the ASF virus appearance in blood and antibodies after an ASFV infection. In addition, it shows the lethality of the different forms of the clinical disease, which ranges from acute to a subacute, as well as from recovered animals. Antibodies are detectable for a long period of time following the initial exposure

**Fig. 11:**
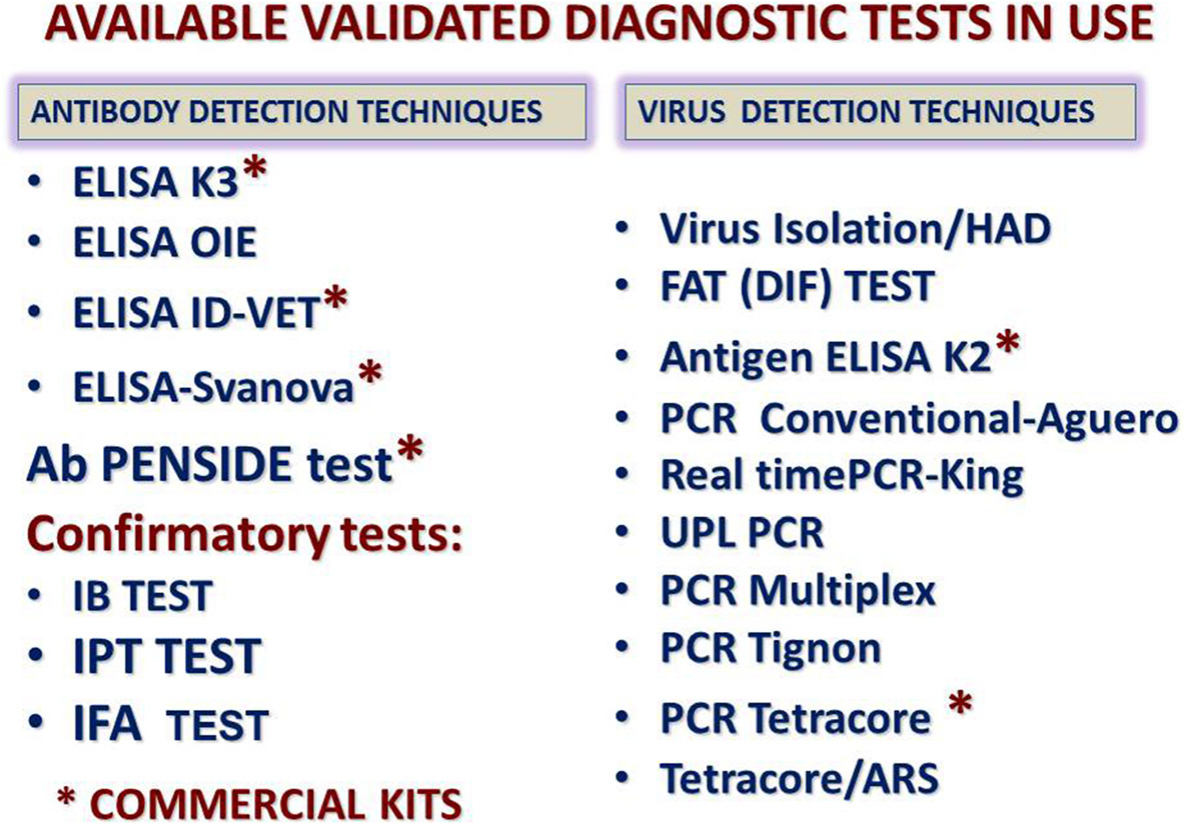
Summary of current available validated ASF Diagnosis tests (source: EURL, INIA-CISA, Valdeolmos, Madrid, Spain). A significant number of tests are available for ASF diagnosis. Since 2010 several new commercial tests have been incorporated in the market, and several others are coming from companies mainly referred to new PCR commercial kit from Ingenasa, Life technologies, or Quiagen that would require to be validated by International Organisms (OIE, EU…)

Early detection of the disease is required to implement sanitary and biosecurity control measures in order to prevent the spread of the disease [49]. However, ASF clinical diagnosis is not easy due to (i) the wide range of clinical forms and the disease’s complex epidemiology, with a number of different scenarios, and (ii) the similarity of its symptoms to those of other viral infections such as CSF, PRRS, swine erysipelas, salmonella, Porcine Dermatitis and Nephropathy Syndrome, as well as other septicaemic conditions such as poisoning. Therefore, rapid laboratory diagnosis is essential and it should involve the detection and identification of the virus particles and the specific anti-ASFV antibodies. A good laboratory diagnosis and interpretation provide relevant information on infection dynamic that will be very helpful to deploy effective control–eradication programs (Fig. 10). The techniques currently in use provide a confident diagnosis of ASF in any epidemiological situation [85–89] (Fig. 11). The use of the most appropriate diagnostic tools updated for all scenarios is critical for successfully implementing effective control programs. PCR tests are the first choice for early detection of the ASFV genome in epidemic situations, although not all such tests are fully sensitive to the low viraemia levels, which can be most often evident in infected animals of endemic zones. For antibody detection, it is important to note that current ELISA tests have a limited sensitivity in the case of low antibody titres and usually detect antibodies from 12–14 days post-infection. Indirect Immunoperoxidase tests (IPT) and Indirect Immunofluorescence tests (IIF) are very useful for detecting specific antibodies, although they cannot be used as screening tests [87]. Once the best and most readily available tools for diagnosis have been identified, it is also necessary to apply a specific value to each diagnostic test and bear in mind their limits in the context of each epidemiological situation, i.e. whether it is taking place in an ASF-free region or an endemic zone, in case of an outbreak. If ASFV enters a disease-free area, virus isolation should be carried out to confirm the presence of the disease. Due to the ASF characteristics and disease dynamics, it is essential that virus and antibody detection techniques be performed in parallel by diagnostic laboratories to ensure a complete picture of the epidemiological situation on a day-to-day basis.

### Vaccines

No vaccine currently exists to prevent and control ASF. For over 40 years various different strategies have been employed in the search for an effective vaccine for this disease. Given the complexity of the virus, with genes involved in the evasion of the immune response [90–92], and the absence of an effective protection mediated by neutralizing antibodies [93], the conventional formulations of live and inactivated vaccines that work with the majority of pathogens have not yet been efficient as protection against ASFV. Nevertheless, it is known that recovered pigs can be protected against subsequent infections with related viruses, as well as partial protection can be achieved with attenuated and low virulent isolates [62, 94, 95]. These data together the advances on the molecular and biological characteristics of the virus [11, 13], and on the immune mechanism that could be involved in protection [92], have led to the development of new promising vaccine candidates. Currently, a number of different approaches are under study [96–100], the most promising of which are based on stimulating the cytolytic CD8+ T-cell and antibody response [92, 98, 99]. These strategies include the construction of deletion mutants from virulent or moderate to low virulent virus isolates, by deleting genes involved in i.e replication, virulence, cellular transport or innate immune response [96, 97, 100]. These vaccine candidates are in a first assessment step, so studies for safety, adverse reactions, potential persistence and transmission in the field are far to be evaluated.

### How to control the disease

Disease knowledge and epidemiological information is crucial for controlling ASF in affected areas. Information about the type of hosts involved, their location, and potential role in virus transmission and spread, the biological characteristics of the circulating virus and the clinical picture to be expected given the host affected, as well as the environmental, social and cultural features of the disease site a definition of risk factors for ASFV entrance and spreading in and specific area, are of major importance and should be elucidated for the provision of an effective control programme.

In areas with limited resources to fight against ASF, education of veterinarians, producers, and farmers is a major issue to maintain a regular clinical inspection of animals as well as the reinforcement of preventive biosecurity measures to guarantee the safe production and marketing of pigs and pig as a means of optimizing control strategies based on risk reduction that would lessen the laboratory costs of contingency and control plans.

### Control-eradication strategies

#### Africa

Currently, regional initiatives exist that include specific control programmes designed to reduce the impact of ASF on the pig sector in Africa. As well, an initiative is being prepared by AU-IBAR-FAO for the control of the disease in eastern Africa. The main strategy is based in controlling ASF in the affected areas and to prevent it from being introduced into non-infected areas. Regional governments and international organisms such as AU-IBAR/FAO working groups are working on ways to improve disease information, identify socio-economic drivers, increase awareness amongst farmers, butchers and other pig-sector stakeholders, strengthen the capacities of veterinary services in disease detection, diagnosis, surveillance, management, contingency planning and emergency response, and pinpoint priority actions and opportunities for collaboration. Disease monitoring and epidemio-surveillance systems are weak and data and information about ASF are mainly being generated by passive surveillance. To guide the establishment of a national surveillance system for ASF, strategies should be developed based on existing FAO and OIE guidelines. The design of risk-based surveillance systems that take into account risk factors for ASF occurrence and transmission will maximize the efficiency and efficacy of the system.

#### Eastern Europe

Non-EU countries are combating ASF and trying to prevent the disease from spreading. However, few effective results have been obtained so far and the disease continues to spread into neighbouring countries, mainly along wild boar corridors, and other ways of virus transmission could occur at any time.

In EU countries, the prevention measures implemented when ASF first appeared in Eastern Europe in 2007 were initially based on strengthening surveillance programmes. Subsequently, as ASF was progressing, a revision of contingency plans in EU member states was carried out that, along with the addition of new methods to the existing preventive measures, enabled the virus to be detected very quickly. As the situation got worse, however, an increase in protection measures was also executed, which currently includes the disinfecting of vehicles, stricter controls at border crossings, the suspension of livestock markets, greater biosecurity on farms, awareness-raising campaigns and tougher vigilance programmes [101–103]. This latter task currently involves an increase in the number of diagnostic analyses of domestic pigs and wild boar, the creation of buffer zones aimed at reducing wild boar densities and limiting their ability to cross frontiers, and, in some cases, the preventative slaughtering of pigs in backyard farms in high-risk areas. Evidence of the EU’s capacity to control and contain this disease is the success it has had on the island of Sardinia, from where, despite its presence for two decades, it has never crossed over onto the European mainland. Right from the start of the outbreaks in the Eastern European Union countries in 2014, the European Commission decided to apply the World Trade Organization’s regionalization principle, in line with the OIE’s international standards. Current control and eradication programmes in the EU contain all the elements that should guarantee their success. Awareness-raising, epidemiological information, strict biosecurity and sanitary measures, coordination between all implicated parts, good communication between sources of field information and diagnostic laboratories, advances in the resolution of problems and the deficiencies that arise, the promotion of necessary epidemiological research in affected areas, and so forth. Nevertheless, if affected countries in Eastern Europe are unable to contain the spread of the disease, the EU must be aware that isolated wild boar cases will continue to occur in border areas of the EU, and that they will have to be combated.

### A worldwide threat

The risk of introduction of ASF into new regions is likely to occur in a near future. The major threat is currently for the East Asian countries that maintain important trade and links with ASF endemic African countries, although the risk coming from Eastern Europe bordering countries is not a trivial matter. China, with the highest pig population of the world, and a large proportion of family and free ranging pigs, could encounter difficulties in controlling ASF in case of an incursion into these production systems. Nor should we forget the worldwide trade and communications by roadways, planes and ships place all regions, and continents at risk.

## Conclusions

ASF is a very complex disease, with complex epidemiology and many different scenarios in which certain hosts, playing different roles, interact with a number of different circulating virus isolates. Since no vaccine is currently available, prevention and control must be based on early detection and strict sanitary measures. Early detection should be based on rapid disease recognition in the field, followed by laboratory diagnosis, that it is essential for disease control. Further knowledge of the disease is necessary for progress in prevention and control-eradication strategies in Africa and Eastern Europe. Continuing education of veterinary services, vets, producers and hunters by training, awareness of the risk factors involved in ASFV entrance and spread, virus-host interactions, virus transmission mechanisms, improved knowledge of the presence of vectors and reservoirs, the development of risk maps and models for ASFV diffusion, and the assessment of the role carrier animals play under different scenarios are just some of the issues that must be tackled. Without the pertinent information, the disease cannot be fought. The current panorama indicates that – unfortunately – we will not be able to ignore African swine fever for some time yet. This is a worldwide threat and all countries must be adequately prepared.
